# Children With Medical Complexity in the Canadian Maritimes: Protocol for a Mixed Methods Study

**DOI:** 10.2196/33426

**Published:** 2022-04-06

**Authors:** Sydney Breneol, Janet A Curran, Marilyn Macdonald, William Montelpare, Samuel A Stewart, Ruth Martin-Misener, Jocelyn Vine

**Affiliations:** 1 School of Nursing Faculty of Health Dalhousie University Halifax, NS Canada; 2 Strengthening Transitions in Care Izaac Walton Killam Health Centre Halifax, NS Canada; 3 Department of Applied Human Sciences University of Prince Edward Island Charlottetown, PE Canada; 4 Department of Community Health and Epidemiology Dalhousie University Halifax, NS Canada

**Keywords:** pediatrics, complex care, health data, mixed methods, children, qualitative, health administration, health care resources, health resources

## Abstract

**Background:**

Ongoing developments in the medical field have improved survival rates and long-term management of children with complex chronic health conditions. While the number of children with medical complexity is small, they use a significant amount of health resources across various health settings and sectors. Research to date exploring this pediatric population has relied primarily on quantitative or qualitative data alone, leaving significant gaps in our understanding of this population.

**Objective:**

The objective of this research is to use health administrative and family-reported data to gain an in-depth understanding of patterns of health resource use and health care needs of children with medical complexity and their families in the Canadian Maritimes.

**Methods:**

An explanatory sequential mixed methods design will be used to achieve our research objective. Phase 1 of this research will leverage the use of health administrative data to examine the prevalence and health service use of children with medical complexity. Phase 2 will use case study methods to collect multiple sources of family-reported data to generate a greater understanding of their experiences, health resource use, and health care needs. Two cases will be developed in each of the 3 provinces. Cases will be developed through semistructured interviews with families and their health care providers and health resource journaling. Findings will be triangulated from phase 1 and 2 using a joint display table to visually depict the convergence and divergence between the quantitative and qualitative findings. This triangulation will result in a comprehensive and in-depth understanding into the population of children with medical complexity.

**Results:**

This study will be completed in May 2022. Findings from each phase of the research and integration of the two will be reported in full in 2022.

**Conclusions:**

There is a current disconnect between the Canadian health care system and the needs of children with medical complexity and their families. By combining health administrative and family-reported data, this study will unveil critical information about children with medical complexity and their families to more efficiently and effectively meet their health care needs. Results from this research will be the first step in designing patient-oriented health policies and programs to improve the health care experiences, health system use, and health outcomes of children with medical complexity and their families.

**International Registered Report Identifier (IRRID):**

DERR1-10.2196/33426

## Introduction

Ongoing developments in the medical field have improved survival rates and management of children with complex chronic health conditions [1,2]. Frequently described as children with medical complexity, these children are often diagnosed with a wide array of pediatric conditions [3,4]. Recognizing the need for conceptual agreeance across clinical and research initiatives to distinguish this unique pediatric population, Cohen et al [3] presented a definitional framework for children with medical complexity. Rather than proposing a diagnosis-specific definition, this framework describes a noncategorical and inclusive approach to conceptualizing medical complexity in children. Cohen et al [3] identified 4 intersecting domains specific to children with medical complexity: (1) the presence of a diagnosed or suspected complex chronic condition, (2) significant family-identified needs, (3) functional limitations that are often severe and may require the use of technological assistance, and (4) high health resource use [3]. This definitional framework is now being used widely to describe this vulnerable and important pediatric group across policy, clinical, and research sectors [4,5].

Literature exploring pediatric complex care has been steadily increasing over the past two decades [1]. Evidence emerging over this time suggests that while children with medical complexity represent a small proportion of the overall pediatric population, they consume a disproportionate amount of health care resources [6,7]. Despite approximately 89% of these children being discharged home from inpatient settings [8], their resource-intensive needs are infrequently met by the current health care system, leaving a substantial burden on families to provide expert medical and supportive care and facilitate care coordination activities for their child. Furthermore, these families often report inadequate support, difficulty accessing services, and other unmet health care needs [9-14]. These findings highlight a clear disconnect between current health care services and the care needs of children with medical complexity and their families.

It is critical that we begin to develop and evaluate family-centered strategies tailored to the needs of this population. However, to achieve this, we must address major knowledge gaps within the literature. First, much of the empirical research has been conducted within the United States health care system [8,13-15], with only 2 main reports published in the last 10 years examining the prevalence and health service use of these children within the Canadian health care system [6,7]. While studies from the United States are informative, it is critical to gain greater regional and jurisdictional understanding of the prevalence, clinical characteristics, health resource use, and health care needs of this population within Canada. Second, the literature exploring this pediatric population primarily relies on routinely collected health administrative data [7,8,15,16]. While this data source has several strengths, such as having access to large population samples across various time frames, there are important limitations to the use of health data to consider. Families use a range of health resources not captured by health administrative data alone (ie, private respite care services, local community-run health programs, private physiotherapy, acupuncture, massage therapy, naturopathic doctors). This leads to a significant gap in our understanding of the true health resource use and care needs of children with medical complexity and their families. Qualitative research methods are designed to explore this gap whereby researchers speak directly to children and families about their lived experience. As such, combining health administrative data with richly descriptive qualitative reports from families is one strategy to fully explore their health resource use and care needs. Using a mixed methods approach could provide researchers, clinicians, families, and decision makers with a detailed and comprehensive understanding of prevalence, clinical characteristics, health resource use, and health care needs of children with medical complexity and their families. Without this information, decision makers may not have all the necessary information to create family-oriented recommendations to support the health of these children and their families.

There remains a significant gap in our understanding of the true extent of health resources used by children with medical complexity and their families living in their home communities. Greater efforts are needed to map health resource use across the public, private, and community sectors to provide the foundational knowledge needed to develop evidence-informed recommendations and strategic directions to support the health and needs of children with medical complexity and their families. As such, the objective of this research is to use health administrative and family-reported data to gain an in-depth understanding into patterns of health resource use and health care needs of children with medical complexity and their families in the Canadian Maritimes (Prince Edward Island [PEI], Nova Scotia [NS], and New Brunswick [NB]). To achieve this objective, the following research questions will be addressed: (1) What are the prevalence and clinical characteristics of children with medical complexity in the Canadian Maritimes? (2) What are the patterns of health care use as described by health administrative data for children with medical complexity? (3) What are the family-reported experiences, health resource use, and health care needs of children with medical complexity and their families? (4) In what ways do the family-reported experiences, care needs, and health resource use converge and diverge with the characteristics and health service use as reported by health administrative data among children with medical complexity and their families in the Canadian Maritime Provinces?

## Methods

To achieve our research aim, an explanatory sequential mixed methods design (quantitative and qualitative) will be used [17].

### Ethics Approval

Ethics approval has been obtained from the Izaac Walton Killam Health Centre research ethics board (#1026835 and #1024934).

### Phase One

#### Design

To understand the prevalence and health service use of children with medical complexity, we will conduct a secondary analysis of routinely collected health administrative data. Access to these data will be obtained through the Health Data Nova Scotia Secure Data Repository Platform and the pediatric tertiary care facility’s decision support services. To achieve this study objective, a 2-phase process will occur. First, discharge data from the Maritimes’ only pediatric tertiary care facility will be used to identify and characterize children with medical complexity in the Maritimes. Next, the health card number of all NS residents identified within the cohort will be linked to the provincial’s health administrative data sets to examine their health resource use.

#### Study Setting

The primary site of this research is the only pediatric tertiary care facility located in the Canadian Maritimes, providing a unique opportunity for multijurisdictional research given their mandate to care for children, youth, and families in all 3 provinces. This site was chosen for this research as children’s hospitals have been identified as the main care site for children with complex chronic health conditions and can provide a representative sample of this population in the 3 provinces [8,18]. Based on the limitations in cross-provincial data linkages, health care resource use will only be explored in NS. NS data is the sole source of health resource use by children with medical complexity and will be relied upon to extrapolate use in the other Maritime Provinces (PEI and NB).

#### Data Sources

Five health care databases will be accessed in this study: the pediatric tertiary care facility’s discharge data, MSI Physician Billings (MED), National Ambulatory Care Reporting System (NACRS), Canadian Institute for Health Information Discharge Abstract Database (CIHI-DAD), Vital Statistics–Death (VITAL).

#### Study Population and Identification

All children and youth aged 0 to 18 years discharged from the pediatric tertiary care facility between April 1, 2004, and March 31, 2014, who meet the definitional framework of Cohen et al [3] for children with medical complexity will be included in the analysis. We know from previous published literature that children with medical complexity are small in number. As such, to ensure a cohort suitable to power a regression analysis, we will examine prevalence over a 10-year time period. This time frame also provides health resource data for up to 5 years (March 2019). The definitional framework of Cohen et al [3] will be operationalized through the application of the Pediatric Medical Complexity Algorithm (PMCA) 3.0 [19]. The PMCA is a validated algorithm to identify and classify the pediatric population based on level of medical complexity within health administrative data [19]. An individual child will only be included once in the cohort. If a child was discharged more than once during the study period, the earliest discharge date with a complex chronic condition will be used as the index date to begin tracking health resource use. Our final cohort will be all children who are classified by the algorithm as children with complex chronic conditions. Health card numbers for the identified sample will be retrieved from the pediatric tertiary care facility’s discharge database by a data analyst and sent to a health system partner organization for encryption to preserve confidentiality. These encrypted health card numbers are then sent directly to the provincial health data repository for linking with MED, NACRS, CIHI-DAD, and VITAL for all NS residents. At no point during this process will the research team have access to the unencrypted or encrypted health card numbers. A 3:1 matched control cohort will be identified by using age, sex, and postal code as matching variables. Matched cases will be used to provide a comparator population and control for potential confounders that may influence health resource use [20,21]. Once the cohort is identified, their health resource use will be followed up to a 5-year period or up to age 18 years.

#### Measures

Variables related to patient demographics will include age, sex, urban/rural residence, organ system involvement, and care team characteristics. Race and ethnicity data were not accessible, as they are not included as routinely collected variables in the health administrative data sets. Variables related to health care use will include inpatient hospital visits, outpatient hospital visits, home care services, emergency department visits, and transfers between care locations. This health data will encompass both tertiary and community care hospitals.

#### Data Analysis

To address our first research objective, the prevalence of children with medical complexity from 2004 to 2014 will be estimated using prevalence rate calculations. The estimated prevalence rate will be obtained by dividing the number of cases of children with medical complexity identified by the PMCA with the total number of children estimated in the Statistics Canada Census Data for Nova Scotia (2016). Prevalence will be further stratified based on age, sex (as assigned at birth), clinical diagnosis category (categorized by the PMCA [19]), and geographical location (urban/rural). Urban and rural residence will be determined by the first 3 digits of their postal code. Age will be analyzed categorically (0-11 months, 1-4 years, 5-9 years, 10-13 years, and 14-18 years). Descriptive statistics will be used to describe the characteristics of children with medical complexity (age, sex, clinical diagnosis category, and urban/rural location).

To address the second research objective, health resource use for both case and control cohorts will be explored using descriptive and inferential statistics. Descriptive statistics including mean (standard deviation), median (interquartile range), and count (percentage) will be used to describe the number of services received, types of medical specialties, and health resource use for children with medical complexity over a 5-year follow-up period. Health resources will be grouped by inpatient admissions, emergency department visits, length of stay, location of care, outpatient services, home care use, and ambulance transfers. Rates of health resource use and length of stay will be further stratified by clinical diagnosis category, age, sex, urban/rural location, and level of health care facility (tertiary/community hospital).

To explore any associations between child characteristics and health system use, a negative binomial regression analysis will be run. The primary outcomes of interest will be counts of hospital readmission as defined by any type of inpatient admissions (ie, intensive care admissions), emergency department visits, and outpatient community services defined as primary care visits, home care services, and clinic services. Predictors of interest are age, geographical location, and sex. Last, to explore the hazard ratios for time to and between health resource use, a Prentice, Williams, and Peterson gap-time model will be used. This will illuminate patterns of health resource use within the identified cohort. All data analysis will be performed using the statistical software program Stata (version 9.3, StataCorp LLC).

#### Anticipated Outputs

There are 3 main outputs from this first phase. First, we will have a detailed description of the prevalence of children with medical complexity in 3 Canadian provinces. Second, we will have an understanding of the formal health service use of this vulnerable population. Third, results from this phase will inform participant recruitment and the development of a theoretically based [22] interview guide for use in phase 2 to capture family-identified health resource use and needs.

### Phase Two

#### Design

A case study design will be used to examine the health resource use and health care needs of children with medical complexity and their families in each of the Maritime Provinces. While the definitional framework for children with medical complexity advances our characterization of this pediatric cohort, there remains little understanding regarding the key health outcomes and their measurability for this population [22]. Case study research is an approach to developing and generating a rich description of complex phenomena in the real-world context and can elicit the answer to how, what, and why questions [23]. For example, how children with medical complexity and their families use the health care system, what types of services are accessed, what gaps and areas for improvement exist, and why these patterns may be occurring. Each case will be informed by 3 sources of data: (1) interviews with families, (2) interviews with individual members of the care team in their home community, and (3) self-reported health resource use.

#### Study Population and Sampling

A purposive sampling strategy [24] will be used to recruit children aged 0 to 18 years matching the Cohen et al [3] definitional framework for medical complexity. We will purposively recruit children and families fitting specific characteristics (eg, demographics, clinical characteristics, level of complexity, health resource use) based on significant findings from phase 1. For example, this may include certain clinical presentations or urban/rural residency that may prompt further examination. Families must be primary residents in one of the provinces of interest (NS, NB, or PEI); 2 cases from PEI, NB, and NS will be developed to capture the potentially varying experiences of children and families living inside and outside of the provincial boundaries of the pediatric tertiary care facility. Families within 1 to 3 months and one family within 2 to 3 years of their initial discharge from hospital will be recruited to explore the experiences of these children and their families at differing points in their care. Children and families must be able to speak the English or French language to be eligible to participate. We will not attempt to specifically recruit participants identified within the phase 1 data set. However, it is anticipated that potential participants in the qualitative phase will have been captured in the health administrative cohort.

The number of participants is not the focus in case study design; rather, it is about gathering multiple forms of data from various perspectives to develop a deeper understanding of a specific case. Two case studies will be developed for each Maritime Province (PEI, NS, and NB), resulting in a total of 6. This will allow for the examination of differing familial and contextual factors. One primary caregiver will self-identify as the primary contact for the study. If more than one caregiver would like to be interviewed, all caregivers will be interviewed at the same time. The primary caregiver will also be asked to identify a maximum of 2 key members of the care team who can be approached for an interview.

Multiple recruitment strategies will be used to reach our target population. Recruitment flyers will be posted to relevant units at the pediatric tertiary care facility, community pediatric care sites, and social media platforms. We will also circulate the recruitment poster and study summary via email to key stakeholders involved in care delivery for children with medical complexity and families to share with their networks. An email and phone number contact for the principal investigator will be on all recruitment materials. The principal investigator will respond to all inquiries related to study participation and will provide potential participants with additional study information and an eligibility screening checklist. Once eligibility is confirmed, the principal investigator will forward the consent form for their review. This consent form will be reviewed with participants prior to the interview and signed.

#### Measure

A semistructured interview guide for families will be developed based on significant findings from phase 1 and the 10 domains of health for children with medical complexity [22]. Barnert et al [22] provided the most comprehensive understanding of the conceptualization of population health for children with medical complexity and their families by creating a conceptual framework outlining 10 domains of health for children with medical complexity. These domains include (1) basic needs, (2) inclusive education, (3) child social integration, (4) child health-related quality of life, (5) long-term child self-sufficiency, (6) family social integration, (7) community system supports, (8) health care system supports, (9) high-quality patient-centered medical home, and (10) family-centered care [22]. This interview guide will also include prompts that are informed by significant findings from phase 1. As case study research is designed to address how, what, and why questions, findings from phase 1 will be used to develop prompts and potential questions to create a more comprehensive understanding of observed and unobserved patterns of service use.

Before family interviews occur, the interview guide will be pilot tested through a think-out-loud session with a parent researcher who works at the tertiary care facility. Changes will be made as required following this pilot testing.

#### Procedure

All interviews will take place over the phone or using the Zoom video conferencing system. Family interviews are anticipated to last 45 to 60 minutes. Demographic and socioeconomic information will be collected on families at the beginning of the interview process. This will include number of individuals in the family unit, child’s health conditions, type of medical device/technology, urban/suburban/rural community, child’s gender (as identified by the child), participant’s gender, child’s age, participant’s age, participant’s race/ethnicity, employment status of the caregivers, and access to transportation services. Families will be asked to identify all of the individuals or specialty clinics involved in the care of their child. An additional data source for the development of the case studies will include asking families to track their health resource use over a 3-week period. This time frame was chosen through consultations with clinicians and researchers, and while we recognize that health resource use can vary greatly among individuals with complex chronic conditions, we did not want study procedures to place unnecessary burdens on families. Families will be provided a health resource journal with a draft template to follow. Within this diary, families will be encouraged to track encounters with services and supports needed to provide care for and support the health of their child. This includes but is not limited to ambulatory care clinic visits, inpatient stays, acupuncture, physiotherapy visits, home care visits, emergency department visits, respite care, and dental visits. Families will also be prompted to track the care coordination activities they undertake (ie, calling different clinics to arrange appointments on the same day). Additionally, we will ask study participants if they believe their 3-week time frame was representative of their average health resource use. A CAD $50 (US $40) gift certificate to either Superstore, Amazon, or Irving Gas will be provided to families in appreciation for their time.

Health care provider semistructured interviews will be composed of 3 to 4 questions developed based upon the respective family interview to reflect specifically on local context and the participating families. Interviews are anticipated to last 10 to 15 minutes over the phone or Zoom video conferencing system. This data will be used to supplement the family-reported experience, providing more context to the identified case. A CAD $10 (US $8) Tim Hortons gift card will be provided to participating care team members.

#### Data Analysis

All interviews will be transcribed verbatim and uploaded to NVivo (version 11, QSR International) qualitative data analysis software. Given the recognized need for theory-informed approaches to case study design [25], all interviews will be coded using a deductive content analysis approach based on the capability, opportunity, motivation–behavior (COM-B) theoretical mode [26]. The COM-B is a comprehensive framework created to explore the interactional factors that influence health behavior [26]. This will allow us to explore the use of health resources, why they might be the way they are, and what resources are required to meet the health care needs of families. Further, this analysis approach could be used in future studies aiming to map study findings to the behavior change wheel to design a knowledge translation intervention [26]. The COM-B will provide an initial theory-based coding scheme to deductively code qualitative findings. Two independent coders will code the 3 domains of capability, opportunity, and motivation. Following this, an inductive coding analysis approach will occur within each domain to group similar statements. This will reveal the presence of contradictory and common themes throughout the data while providing a theoretical foundation that can help better understand the phenomenon under investigation. Self-reported health resource data from the family health resource journaling will be examined using descriptive and frequency statistics (mean, median, range, count).

Interview and self-reported data for each case will undergo data triangulation to create a greater understanding of child and family experiences. All quantitative and qualitative data will be organized into a matrix table based on themes resulting from the interview and self-reported heath resource data to examine patterns of convergence and divergence within each case study [23,25]. Descriptive statistics will be used to describe the variables captured in the self-reported diaries. Each case will be analyzed separately to create an in-depth representation of their individual experiences. Member-checking will occur with the findings from each case by presenting the results back to the family to check that we captured their experiences accurately [24]. After data analysis is completed for each case study, a cross-case analysis will occur to examine common themes and patterns and areas of divergence among cases [23]. A matrix table will be created to display data from each individual case based on common and emergent themes [23]. This matrix will reveal patterns or uniqueness among cases [23].

#### Anticipated Outputs

There are 2 main outputs from this phase of the research. First, we will have a rich description of the first-hand experiences of children with medical complexity and their families, as well as their formal and informal health resource use. Second, we will use this family-reported data to compare it with findings from phase 1 to develop a greater and more comprehensive understanding of the health resource use and health care needs of children with medical complexity and their families.

#### Data Triangulation

The intent of data integration in an explanatory sequential design is to examine the extent to which the follow-up rich qualitative results connect or explain the initial quantitative data [17]. To do this, we will triangulate phase 1 and phase 2 data using a joint display table to visually depict the quantitative and qualitative phases. This joint display will be organized based on a statistic-by-theme framework, linking relevant and related health administrative data with the follow-up case study findings [17]. Using this data triangulation approach, we will be able to create a greater understanding of the population, health resource use, and health care needs of children with medical complexity in the Canadian Maritime provinces.

## Results

Phase 1 and phase 2 are in progress. Findings from each phase of research and the integration of the two will be reported in full in 2022.

## Discussion

### Principal Considerations

There is a current disconnect between the Canadian health care system services and the needs of children with medical complexity and their families [1]. By combining both health administrative and family-reported data, this study can unveil critical information about children with medical complexity and their families to health researchers, clinicians, policy makers, administrators, and families themselves. Mixed methods research has been underused in the current literature surrounding pediatric complex care, leaving gaps in our understanding of the responsiveness of our health care services caring for this vulnerable population. Current literature generally focuses on hospital-based health service use, such as emergency department encounters and inpatient admissions, with limited exploration into home and community-based resources [5,7,8]. This is of particular importance given the growing shift in care provision from hospital to community-based care for individuals living with medical complexity [1,8]. As such, this research study is designed to take a novel approach to the study of children with medical complexity and their health service use, contributing to the advancement of this body of research. We anticipate that this work will increase our understanding of the extent of health resources used and needed by children with medical complexity and their families to support their health and well-being while living in their home communities. While the development of an intervention is beyond the scope of this proposed research, the strong theoretical underpinning, methodology, and methods used will ensure its findings can be used in future work to advocate for and inform the design of health policy and programs in the Canadian Maritimes for this population of children and families. As such, this research has the potential to improve the health care delivery, experiences, and outcomes for children with medical complexity and their families.

### Limitations

The findings from this research should be considered with the following limitations in mind. A limitation to secondary data analysis is that the researcher can only work with the data originally collected and stored. Hospital data are collected primarily for administrative purposes and are not specifically designed for research [20]. This can lead to incomplete or missing records and variability in diagnostic codes [20]. Further, although the inclusion criteria to identify the cohort of children with medical complexity has been used in previous studies [27-30], there are limitations to relying solely on diagnostic codes. The use of diagnostic codes may result in patients with medical complexity not being captured or capturing those who would not fit the definitional framework. Furthermore, we make the assumption that children with medical complexity will have received care at the pediatric tertiary care facility at least once during their initial or follow-up medical care. As such, our prevalence estimates may be slightly underrepresented given the possibility that some of these children may be seen and managed fully by their local/regional hospitals. We are also limited by the lack of sociodemographic variables, such as race and ethnicity, available in our health administrative data sets. We strongly believe, however, that these are critical intersectional factors in the lives of these families and require exploration in future work. Furthermore, not all community hospitals report to NACRS at the highest level, resulting in potentially incomplete reporting related to emergency department transfers. It is also important to note that due to constraints across provincial data linkage, we chose to use NS and their provincial health administrative databases as the exemplary province to explore health resource use for children with medical complexity. We recognize that health resource use as indicated by health administrative data may differ in PEI and NB.

Although purposive sampling will be used for phase 2 to explore results found during phase 1, participants’ opinions or experiences may not be shared by other families or health care professionals. This study will also be limited to the experiences of children from one pediatric tertiary care center serving children and families in 3 small provinces in Eastern Canada that operates within a publicly funded health care system. Other health centers may differ in the structure, programs, and care provision for children with medical complexity; thus, results may not be reflective of other families and sites.

### Conclusion

Improvements in medical treatments and technologies will likely result in an increased population of children with complex conditions. It is critical that we begin to develop a greater understanding of the health resource use of this vulnerable population to more efficiently and effectively meet their health care needs. Results from this research will be an important step forward in designing patient-oriented health policies and programs to improve the health care experiences, health system use, and health outcomes of children with medical complexity and their families.

## Abbreviations

CIHI-DAD: Canadian Institute for Health Information Discharge Abstract Database
COM-B: capability, opportunity, motivation–behavior
MED: MSI Physician Billings
NACRS: National Ambulatory Care Reporting System
NB: New Brunswick
NS: Nova Scotia
PEI: Prince Edward Island
PMCA: Pediatric Medical Complexity Algorithm
VITAL: Vital Statistics–Death
